# Characterization of Antibiotic Resistance Gene Abundance and Microbiota Composition in Feces of Organic and Conventional Pigs from Four EU Countries

**DOI:** 10.1371/journal.pone.0132892

**Published:** 2015-07-28

**Authors:** Lenka Gerzova, Vladimir Babak, Karel Sedlar, Marcela Faldynova, Petra Videnska, Darina Cejkova, Annette Nygaard Jensen, Martine Denis, Annaelle Kerouanton, Antonia Ricci, Veronica Cibin, Julia Österberg, Ivan Rychlik

**Affiliations:** 1 Veterinary Research Institute, Brno, Czech Republic; 2 Brno University of Technology, Brno, Czech Republic; 3 Technical University of Denmark, National Food Institute, Copenhagen, Denmark; 4 Anses, Hygiene and Quality of Poultry and Pig Products Unit, Ploufragan, France; 5 Istituto Zooprofilattico Sperimentale delle Venezie, Padova, Italy; 6 National Veterinary Institute (SVA), Uppsala, Sweden; University Medical Center Utrecht, NETHERLANDS

## Abstract

One of the recent trends in animal production is the revival of interest in organic farming. The increased consumer interest in organic animal farming is mainly due to concerns about animal welfare and the use of antibiotics in conventional farming. On the other hand, providing animals with a more natural lifestyle implies their increased exposure to environmental sources of different microorganisms including pathogens. To address these concerns, we determined the abundance of antibiotic resistance and diversity within fecal microbiota in pigs kept under conventional and organic farming systems in Sweden, Denmark, France and Italy. The abundance of *sul1*, *sul2*, *strA*, *tet(A)*, *tet(B)* and *cat* antibiotic resistance genes was determined in 468 samples by real-time PCR and the fecal microbiota diversity was characterized in 48 selected samples by pyrosequencing of V3/V4 regions of 16S rRNA. Contrary to our expectations, there were no extensive differences between the abundance of tested antibiotic resistance genes in microbiota originating from organic or conventionally housed pigs within individual countries. There were also no differences in the microbiota composition of organic and conventional pigs. The only significant difference was the difference in the abundance of antibiotic resistance genes in the samples from different countries. Fecal microbiota in the samples originating from southern European countries (Italy, France) exhibited significantly higher antibiotic resistance gene abundance than those from northern parts of Europe (Denmark, Sweden). Therefore, the geographical location of the herd influenced the antibiotic resistance in the fecal microbiota more than farm’s status as organic or conventional.

## Introduction

A recent trend in animal production is the revival of interest in organic farming [1,2]. Although this trend is mainly motivated by animal welfare, concerns about the use of antibiotics in conventional farming systems also contributed to increased interest in organic farming [3–5]. On the other hand, organically farmed animals have access to the outdoor environment with harbors different sources of viral, bacterial and protozoan microbiota. This may increase the diversity of microbiota in various organs of organic animals including an increased risk of infections by pathogens [6]. The pros and cons of conventional and organic farming therefore raise extensive debates.

Pigs are predominantly reared in intensive production systems where the administration of antibiotics is commonly used to control various diseases [7]. However, every administration of antibiotics leads to the positive selection of antibiotic resistant bacterial clones [8,9]. In contrast to conventional systems, the use of antibiotics is strictly regulated in organic farming and a lower prevalence of antibiotic resistance in microbiota of pigs raised by organic farming methods can therefore be expected.

The quantification of antibiotic resistance in a given bacterial population is commonly performed by culture. However, this approach is limited to the testing method which specifically selects for certain antibiotics and bacterial species. Moreover, it is uncertain whether the presence of antibiotic resistance in certain bacterial species is representative of the entire bacterial population. This is why culture independent techniques, *e*.*g*. real-time PCR quantification or next generation sequencing, have been used to quantitatively assess antibiotic resistance [9–12].

In this study we compared the abundance of selected antibiotic resistance genes and microbial diversity in the feces of organic and conventional pigs. We hypothesized that the abundance of antibiotic resistance genes would be higher in the conventionally grown pigs due to more frequent antibiotic use while microbial diversity might by higher in organic pigs due to their higher exposure to environmental microbiota. Unlike our expectations, there were no significant differences between the prevalence of antibiotic resistance genes or microbiota composition in organic or conventional pigs. However, microbiota in the samples originating from southern Europe exhibited a higher abundance of antibiotic resistance genes than the samples from northern Europe.

## Material and Methods

### Ethics statement

Sampling at slaughter houses were performed under the supervision and permission of the district veterinary officer and pig owners. The owners of the farms were aware that the droppings were being collected for this study and gave their permission for doing so. However, the owners of the slaughterhouses and the individual farmers both gave their permission for sample collection and processing assuming their identity will not be revealed. Due to these confidentiality agreements, the specific locations of the farms and slaugterhouses therefore cannot be disclosed. None of the pigs were slaughtered specifically for research purposes and no other type of samples, e.g. different tissues, were collected from the pigs.

### Sample collection and DNA isolation

In total, 468 intestinal or fecal samples were collected from pigs throughout 2012–2013. Out of these, 233 samples originated from pigs kept under conventional conditions in France (n = 36), Italy (n = 122), Sweden (n = 36) and Denmark (n = 39), and 235 samples were collected from pigs reared in an organic farming system in the same countries, *i*.*e*. in France (n = 42), Italy (n = 116), Sweden (n = 35) and Denmark (n = 42). Samples from French, Swedish and Danish pigs were collected from the colon of slaughtered animals and Italian samples were collected from fresh fecal droppings in pens with finishing pigs. All the samples were stored at -20°C until DNA purification. DNA was extracted using QIAamp DNA Stool Mini kit following the manufacturer’s instructions (Qiagen) and the quality and concentration of the purified DNA was determined spectrophotometrically. DNA was stored at -20°C until further analysis.

### Real-time PCR quantification of antibiotic resistance genes

Real-time PCR was used for the quantification of *sul1*, *sul2*, *strA*, *tet(A)*, *tet(B)* and *cat* genes, as described previously [9,13]. Target antibiotic resistance genes were selected to represent those commonly found in the genomes of gut microbiota [9, 14]. PCR was performed in 3μl volumes in 384-well microplates using QuantiTect SYBR Green PCR Master mix (Qiagen). Dispensing of the PCR master mix, primers, water and DNA was performed using Nanodrop pipeting station (Innovadyne). PCR and signal detection was performed with a LightCycler II (Roche) with an initial denaturation at 95°C for 15 min followed by 40 cycles of denaturation at 95°C for 20 s, primer annealing at 60°C for 30 s and extension at 72°C for 30 s. Each sample was subjected to real-time PCR in triplicate and the mean values of the triplicates were used for subsequent analysis. Amplification of the 16S rRNA gene using domain *Bacteria* specific primers was used as a reference to determine the total amount of bacterial DNA in each sample (for primer sequences see S1 Table). Ct values of genes of interest were subtracted from the Ct value of bacterial 16S rRNA gene amplification (ΔCt) and the relative abundance of each gene of interest was finally calculated as 2^-ΔCt^.

### Comparison of microbiota composition in organic and conventional pigs by pyrosequencing 16S rRNA genes

Five samples from each country and each farming condition were selected for microbiota characterization. The samples were selected to include one sample with extremely high antibiotic resistance gene prevalence, one with extremely low antibiotic gene prevalence and remaining 3 samples were chosen as having average antibiotic resistance gene abundance for each particular country and farming condition. The sixth sample for each country and farming conditions was generated by pooling all available samples from the same country and farming conditions. Altogether 48 DNA samples were used as a template in PCR amplifying over V3/V4 variable region of 16S rRNA genes [12]. PCR was performed using a HotStarTaq *Plus* Master Mix Kit (Qiagen) with initial denaturation at 95°C for 15 min followed by 30 cycles of denaturation at 94°C for 40 s, primer annealing at 55°C for 45 s and extension at 72°C for 1 min. The resulting PCR products were separated on a 1.2% agarose gel and extracted from the gel with a QIAquick Gel Extraction kit (Qiagen). The purified PCR products were subjected to library preparation using a GS Amplicon Library Preparation Kit (Roche), emulsion PCR and pyrosequencing with GS Junior Titanium kits strictly following the manufacturer's instructions (Roche). The pyrosequencing was performed with a GS Junior 454 sequencer (Roche).

The data obtained were processed as described previously [12]. Briefly, fasta and qual files generated as an output of the pyrosequencing were uploaded into Qiime software [15]. Trimming criteria consisted of none or one mismatch in primer sequences and no mismatch in MID sequences. Sequences with a quality score higher than 20 were shortened to the same length of 350 bp and classified with RDP Classifier and RDP Seqmatch with an OTU discrimination level set to 97%. Diversity analyses were performed using both all sequences available for each sample and the same number of randomly selected sequences adjusted to the number of sequences available for the sample with the lowest coverage. The raw sequence reads have been deposited in the NCBI Short Read Archive under the accession number SRP049609.

### Statistics

To achieve normal distribution and to stabilize the variance, real-time PCR data from the abundance of antibiotic resistance genes were transformed using logarithmic transformation and Box-Cox transformation, respectively. In the next step, data were analyzed by ANOVA followed by Tukey HSD post-hoc test (Statistica v.12.0, module ANOVA). Transformed data were also analyzed by principal component analysis (PCA; Statistica v.12.0, module Multivariate exploratory techniques).

## Results

### Real-time PCR quantification of antibiotic resistance genes

**Fig 1:**
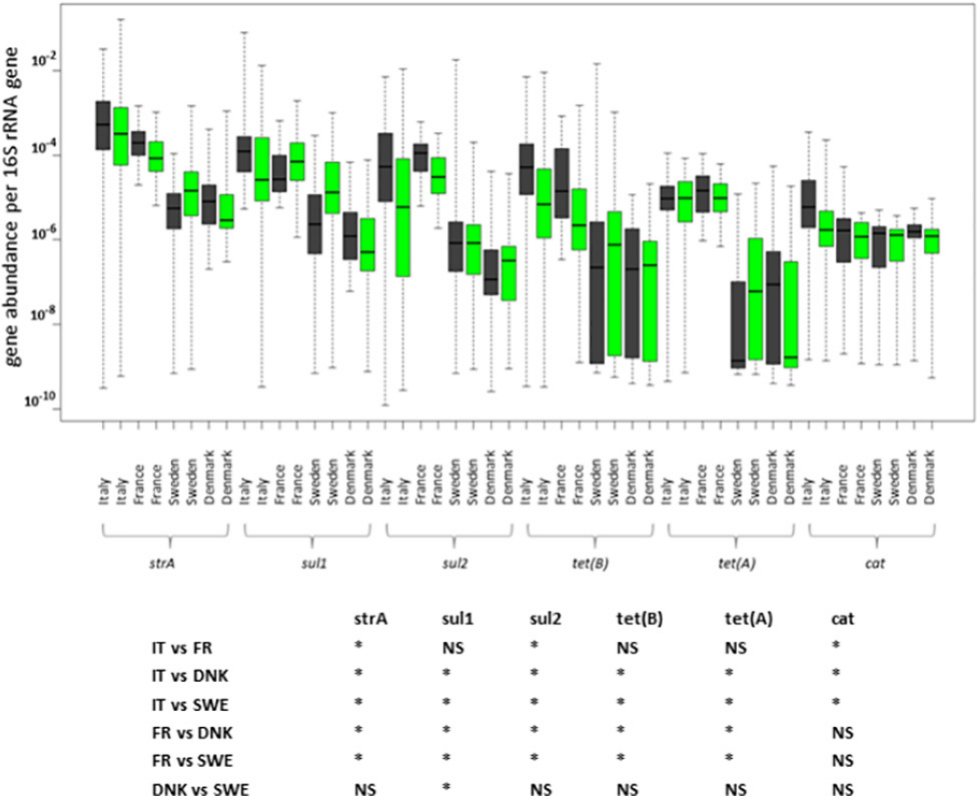
Abundance of antibiotic resistance genes in fecal microbiota of conventional and organic pigs. Antibiotic resistance gene abundance is presented as median, 25th and 75th percentiles (box) and the whiskers indicating the minimal and maximal values recorded. Black boxes, abundance in the microbiota from conventional pigs, green boxes, abundance in microbiota from organic pigs. As shown in the table embedded within the figure, country of sample origin (IT–Italy, FR–France, DNK–Denmark, SWE–Sweden) was a significant source of variation (ANOVA followed by Tukey HSD post-hoc test, P<0.01, asterisks indicate significant difference, NS–Non Significant). On the other hand, comparison of antibiotic resistance gene abundance in feces of pigs from organic and conventional farms within the same country reached statistical difference only for *sul2*, *tet(B)* and *cat* abundance in Italian samples (ANOVA followed by Tukey’s HSD post-hoc test P<0.01).

*strA*, *sul1* and *sul2* genes were the most abundant antibiotic resistance genes in porcine fecal microbiota, independent of country or farming conditions. The median abundance of *strA*, *sul1* and *sul2* genes in samples from Italy, France and Denmark ranged from 10^−4^ to 10^−5^ which means that these genes were present in 1 out of 10^4^ or 10^5^ bacteria forming the total fecal microbiota. *tet(A)* and *tet(B)* genes were present at similar median abundance 10^−5^ in Italian and French samples and at 2–3 logs lower abundance in samples from Denmark and Sweden. The *cat* gene was the least abundant as its abundance ranged around 10^−6^ irrespective of the country of origin or farming conditions (Fig 1). The antibiotic resistance gene abundance was usually higher in samples from Italy and France followed by Denmark and Sweden. This pattern was observed for *strA*, *sul1*, *sul2*, *tet(A)* or *tet(B)* genes. The *cat* gene had quite a similar abundance in all the samples, irrespective of country of origin.

**Fig 2:**
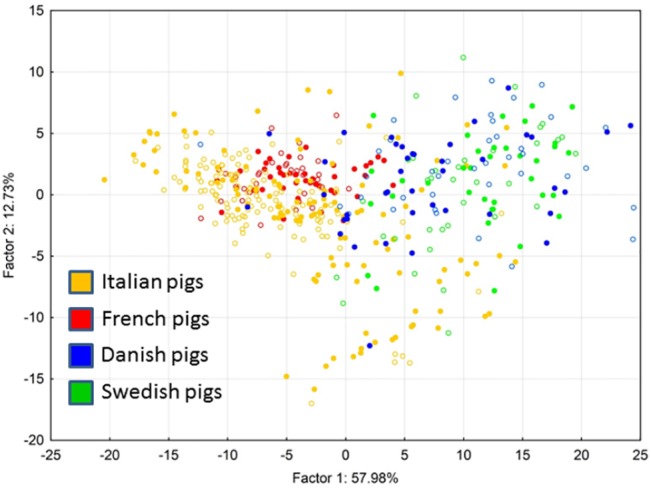
PCA clustering based on the antibiotic resistance gene abundance. Each spot represents an individual sample tested in this study and clustered according to the abundance of 6 antibiotic resistance genes. Factor 1, accounting for nearly 58% of the variation negatively correlated with abundance of *strA*, *sul1*, *sul2*, *tet(A)* and *tet(B)* (correlation coefficients ranged from -0.84 to -0.76). Orange—samples from Italian farms, red—samples from French farms, blue—samples from Danish farms, green—samples from Swedish farms. Opened symbols–samples from pigs kept in conventional farms. Closed symbols–samples from pigs kept in organic farms.

The fact that samples originating from different countries exhibited different antibiotic resistance gene abundance was further supported by PCA clustering which grouped samples from Italy and France, and Denmark and Sweden into 2 different clusters (Fig 2). However, when different farming conditions were compared within the same country, neither the antibiotic resistance gene abundance shown in Fig 1 nor the PCA clustering shown in Fig 2 indicated any differences between the production systems.

### Microbiota composition determined by 16S rRNA pyrosequencing

Since there were differences in the abundance of antibiotic resistance genes in samples originating from different countries, we subsequently examined whether this was caused by the presence of different microbiota members. Furthermore, in this experiment we addressed an original aim of this study, i.e. whether there are differences in microbial diversity in feces of organic and conventional pigs.

Altogether 323,887 sequences were obtained in this study and the number of sequences per sample ranged from 1,837 to 15,706 (S2 Table). Altogether, representatives of 21 phyla were detected throughout the study using RDP Classifier and these were classified into 124 or 117 families when using RDP Classifier or RDP SeqMatch, respectively.

The dominating phyla in conventional porcine fecal samples included *Firmicutes* (74.6 ± 14.5%), *Bacteroidetes* (19.8 ± 11.8%) and *Proteobacteria* (3.39 ± 5.49%). Other phyla were present in less than 1% of total microbiota. At family level, the samples from conventional pigs were dominated by *Ruminococcaceae* (17.55 ± 9.05%), *Lactobacillaceae (*12.99 ± 10.95%*)*, *Clostridiaceae* (11.66 ± 11.31%*)* and *Lachnospiraceae* (10.88 ± 4.84%) followed by *Prevotellaceae*, *Porphyromonadaceae* and *Peptostreptococcaceae* being present at 9.43 ± 8.68%, 7.54 ± 3.49% and 5.83 ± 4.53%, respectively. An additional 6 families were present at 1–5% of all microbiota (S3 Table).

**Fig 3:**
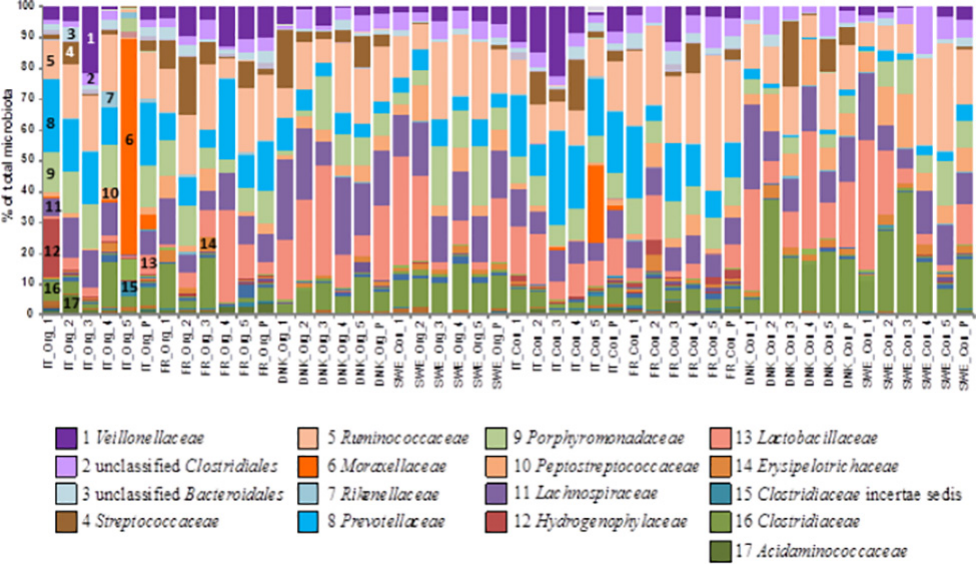
Composition of fecal microbiota of conventional and organic pigs in four countries at family level. Each color represents a particular bacterial family and the following numbers refer to the defined color codes. 1 –*Veillonellaceae*, 2—unclassified *Clostridiales*, 3—unclassified *Bacteroidales*, 4 –*Streptococcaceae*, 5 –*Ruminococcaceae*, 6 –*Moraxellaceae*, 7 –*Rikenellaceae*, 8 –*Prevotellaceae*, 9 –*Porphyromonadaceae*, 10 –*Peptostreptococcaceae*, 11 –*Lachnospiraceae*, 12 –*Hydrogenophylaceae*, 13 –*Lactobacillaceae*, 14 –*Erysipelotrichaceae*, 15—*Clostridiaceae* incertae sedis, 16 –*Clostridiaceae*, 17 –*Acidaminococcaceae*. IT–Italy, FR–France, DNK–Denmark, SWE–Sweden. Org–samples from organic farms, Con–samples from conventional farms. Sample 1–5 for each country and production system represent samples taken from 5 randomly selected pigs. The sixth samples (“P”) always represent pooled samples from particular country and production system.

Samples from organic farming were also dominated by *Firmicutes* (69.5 ± 16.4%) followed by *Bacteroidetes* (22.3 ± 10.0%), *Proteobacteria* (5.75 ± 14.13%) and *Actinobacteria* (1.36 ± 0.96%). At family level, the samples from organic pigs were dominated by *Ruminococcaceae* (15.52 ± 5.61%), *Lachnospiraceae* (13.21 ± 6.21%) and *Lactobacillaceae* (12.73 ± 11.29%), *Prevotellaceae* (9.72 ± 6.69%) followed by *Porphyromonadaceae* (9.24 ± 4.88%) and *Clostridiaceae* (7.84 ± 5.13%). Additional 9 families were present at 1–5% of all microbiota (S3 Table). Rather unique was the presence of *Moraxellaceae* in 3 samples from Italian pigs, two of them originating from organic production and one from a pig at a conventional farm (Fig 3). In addition, we noticed a lower abundance of *Prevotellaceae* and a higher abundance of *Clostridiaceae* in microbiota of conventional pigs from Sweden and Denmark in comparison with all the remaining samples (Fig 3). Apart from the differences above, the fecal microbiota of pigs from different countries and production systems were highly uniform and the only significant difference between microbiota of organic and conventional pigs was recorded in *Bifidobacteriaceae* (for PCoA plots see S1 Fig). *Bifidobacteriaceae* were 3× more frequent in microbiota from organic pigs compared to conventional pigs. However, their total abundance was quite low accounting for around 0.2–0.6% of total microbiota.

## Discussion

In this study we investigated the differences in antibiotic resistance gene abundance and fecal microbiota composition in pigs from organic and conventional production systems originating from 4 different European countries. Rather unexpectedly, there were no extensive differences in antibiotic gene abundance in fecal microbiota from organic and conventional pigs. This was different from reports in poultry in which the abundance of antibiotic resistant *Enterobacteriaceae* was repeatedly higher in chickens from conventional systems than in organic systems [3,16,17] although a recent report disputed the original observations [18]. On the other hand, our findings are similar to the observations in cattle in which the abundance of antibiotic resistant bacteria was also the same in animals originating from organic and conventional production systems [4,19,20]. This conclusion can be explained by the high ability of microbiota to recover following antibiotic therapy [9,12,21,22]. Short therapeutic events early in the production of pigs thus may be of low consequence for the presence of antibiotic resistant bacteria in fecal microbiota of pigs at the time of slaughter. The absence of differences in antibiotic resistance gene abundance by real-time PCR quantification may also reflect that this approach is suitable for the characterization of more extensive differences, whereas smaller differences cannot be discriminated sufficiently. This is supported by a parallel examination of the same samples for the antibiotic resistance in *Escherichia coli* which showed significantly lower levels of antibiotic resistance in *E*. *coli* from organic pigs compared to conventional (data not shown).

Despite the absence of differences in the abundance of antibiotic resistance genes in the microbiota of conventional and organic pigs within the same country, we observed significant differences between countries, except for the resistance to chloramphenicol. Rather similar abundances in the chloramphenicol resistance *cat* gene can be explained by a ban of chloramphenicol use in animal production in the EU in 1994 [23]. On the other hand, the most extensive differences in antibiotic resistance gene abundances among countries were observed for *tet*(A) and *tet*(B) genes, both coding for efflux pump proteins. It is possible that the presence of these efflux pump proteins in the absence of antibiotic pressure results in negative fitness and negative selection of bacteria harboring these genes from the total population [24, 25].

As mentioned above, these country-specific effects are also unlikely to be caused by recent antibiotic therapy since pigs in organic production can be treated only once during a six month period and no antibiotic treatment is allowed in either production systems at the end of the rearing period. Considering the rapid recovery of microbiota after antibiotic withdrawal [9,12,21,22,26], the direct effect of recent antibiotic administration is therefore unlikely. Whether the explanation might be due to the differences in general use and handling of antibiotics among countries would require more solid data on antibiotic use in EU countries [27, 7], both in animal husbandry and in humans. However, a similar, though not that extensive, south/north country effect in antibiotic resistance gene abundance in gut microbiota was observed also in poultry [28].

The composition of pig fecal microbiota observed in this study was similar to that reported by Mulder et al. [29]. Minor differences can be explained by the fact the Mulder et al. tested microbiota composition in 56-day-old piglets while we tested microbiota in adult pigs and age is a known factor affecting gut microbiota composition [30–32]. In addition, ileal and fecal microbiota were characterised in the study of Mulder at al., whereas this paper presents data on colonic or fecal microbiota. Despite this, the absence of extensive differences between gut microbiota of conventional and organic pigs was similar to the absence of significant differences in the composition of microbiota of indoor and outdoor kept pigs reported earlier [29]. We also noticed that when compared with humans or chickens, the microbiota of pigs contained much lower counts of *Bacteroidaceae* [29–33].

*Bifidobacteriaceae* (phylum *Actinobacteria*) was the only family which was significantly increased in microbiota of organic pigs in comparison to conventional ones. Although we do not have any experimental evidence to explain this, representatives of *Bifidobacteriaceae* are capable of digesting different oligosaccharides [34,35]. Organic pigs may have increased access to environmental oligosaccharides of plant origin in comparison to conventional pigs and these oligosaccharides may act as prebiotics positively selecting for *Bifidobacteriaceae* mainly in outdoor, organic pigs.

In this study we showed that the composition of pig gut microbiota is quite conserved and did not differ between conventional and organic pigs or among countries. In addition, there were no differences in selected antibiotic resistance gene abundance in microbiota from conventional or organic pigs from the same country. However, we observed a considerable “country” effect on antibiotic resistance gene abundance which increased from northern to southern Europe. This indicates that the geographical location in which pigs are kept and grown is dominant over the farming system. A recent report on antibiotic use in different EU countries shows that antibiotics, including those against which the genes responsible for resistance were quantified in this study, are used more frequently in farm animal production in France and Italy than in Denmark or Sweden [36]. However, one must be cautious in drawing conclusions from the rather moderate differences found between antibiotic use in Denmark and France. Whether there is an association between general antibiotic policies adopted in human and veterinary medicine in different countries and the abundance of antibiotic resistant bacteria in pig gut microbiota will therefore have to be determined by additional independent studies.

## Supporting Information

S1 Fig Weighted and unweighted PCoA plots based on 16S rRNA gene sequencing of selected samples. No differences based on the country of sample origin or faming conditions were observed. Orange—samples from Italian farms, red—samples from French farms, blue—samples from Danish farms, green—samples from Swedish farms. Opened symbols–samples from pigs kept in conventional farms. Closed symbols–samples from pigs kept in organic farms.
(PDF)

S1 Table List of primers used in this study. (XLS)

S2 Table Basic pyrosequencing characteristics. This file contains basic information on sequence coverage of individual samples together with different indices characterizing the alpha and beta diversities.
(XLS)

S3 Table List of all OTUs identified in this study. List of all OTU identified in this study and classified with RDP classifier to the most probable taxons.
(XLS)
