# Supplementary material for: Characterization of Antibiotic Resistance Gene Abundance and Microbiota Composition in Feces of Organic and Conventional Pigs from Four EU Countries
Source: PLoS One. 2015 Jul 28;10(7):e0132892. doi: 10.1371/journal.pone.0132892 (PMC4517930; doi:10.1371/journal.pone.0132892)
Supplement: S1 Fig — No differences based on the country of sample origin or faming conditions were observed. Orange—samples from Italian farms, red—samples from French farms, blue—samples from Danish farms, green—samples from Swedish farms. Opened symbols–samples from pigs kept in conventional farms. Closed symbols–samples from pigs kept in organic farms. (PDF) [file pone.0132892.s001.pdf]

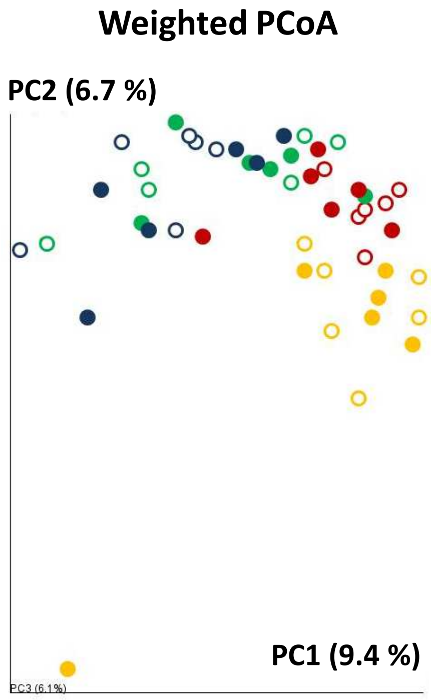

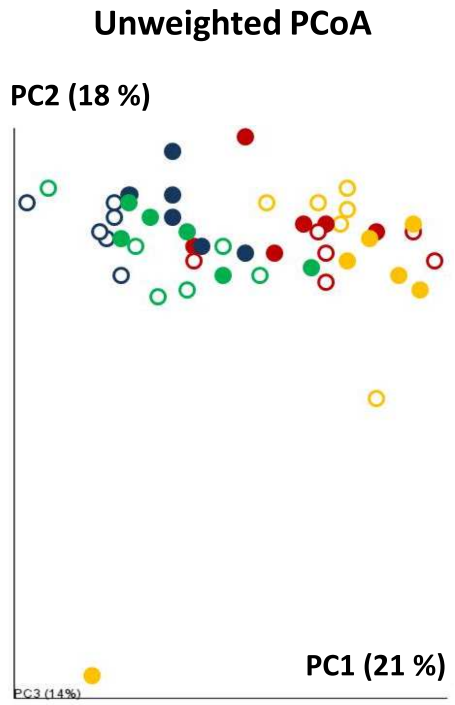

S1 Figure. Weighted and unweighted PCoA plots based on 16S rRNA gene sequencing of selected samples. No differences based on the country of sample origin or faming conditions were observed.
Orange - samples from Italian farms, red - samples from French farms, blue - samples from Danish farms, green - samples from Swedish farms. Opened symbols – samples from pigs kept in conventional farms. Closed symbols – samples from pigs kept in organic farms. Mind quite low PC1 values in both weighted and unweighted PCoA.
